# Which trial do we need? A global, adaptive, platform trial to reduce death and disability from tuberculous meningitis

**DOI:** 10.1016/j.cmi.2023.03.021

**Published:** 2023-03-22

**Authors:** Guy E. Thwaites, James Watson, Nguyen Thuy Thuong Thuong, Julie Huynh, Timothy Walker, Nguyen Hoan Phu

**Affiliations:** 1 Oxford University Clinical Research Unit, Ho Chi Minh City, Viet Nam; 2 Centre for Tropical Medicine and Global Health, Nuffield Department of Medicine, University of Oxford, Oxford, United Kingdom

**Keywords:** Adaptive trial, Global, Platform trial, Randomized controlled trial, Tuberculous meningitis

Before the advent of anti-tuberculosis drugs in the 1940s, tuberculous meningitis (TBM) was a much-feared and almost universally fatal form of tuberculosis. Thus, when the first antituberculosis drugs, streptomycin and para-aminosalycilic acid, became available, they were given first to those with TBM. Suddenly, the disease became treatable. Case fatality fell from 70% with streptomycin and para-aminosalycilic acid, to approximately 30% with the combination of isoniazid, pyrazinamide, and rifampicin [1].

Sadly, the progress has slowed over the last 30 years. TBM case fatality is stuck at approximately 25% in individuals who are HIV-negative and up to 50% in those living with HIV, and nearly everyone dies or is left disabled if the disease is caused by multidrug resistant *Mycobacterium tuberculosis* [2]. Interventions to improve these depressing figures fall into those that enhance bacterial killing, usually by optimizing intra-cerebral anti-tuberculosis drug exposures, and those that control brain inflammation, which has long been recognized as a major contributor to TBM-related death and neurological disability.

Sadly, the progress has slowed over the last 30 years. TBM case fatality is stuck at approximately 25% in individuals who are HIV-negative and up to 50% in those living with HIV, and nearly everyone dies or is left disabled if the disease is caused by multidrug resistant *Mycobacterium tuberculosis* [2]. Interventions to improve these depressing figures fall into those that enhance bacterial killing, usually by optimizing intra-cerebral anti-tuberculosis drug exposures, and those that control brain inflammation, which has long been recognized as a major contributor to TBM-related death and neurological disability.

Over the last 2 decades, a small number of randomized controlled trials have been conducted aiming at improving TBM outcomes [2]. The interventions tested included higher doses of rifampicin (15e35 mg/kg) [3], adding anti-tuberculosis drugs with good brain penetration (e.g. fluoroquinolones and linezolid) [4], or controlling brain inflammation with adjunctive corticosteroids and aspirin [5]. However, with the exception of adjunctive corticosteroids, which reduced death (but not disability) in HIV-negative individuals with TBM [6], the trials of the last 20 years have not established new, outcome-improving, changes in treatment.

The lack of success may be explained by the relative rarity of TBM, the difficulties of confirming the diagnosis, the challenges of enrolling very unwell patients into trials, and limited interest from funding agencies. In general, the trials conducted have been too small (<800 participants), too slow to recruit (often just 1 or 2 centres), and have only investigated 1, sometimes 2, interventions at a time. Furthermore, there are currently three active trials investigating the same intervention (high-dose rifampicin) [2], suggesting potential duplication of effort.

In contrast to the modest achievements of TBM research over the last 2 decades, the wider advances in treating pulmonary tuberculosis have been spectacular. A resurgent anti-tuberculosis drug pipeline and associated clinical trials have produced an entirely new, oral, highly effective 6-month treatment regimen (bedaquiline, pretomanid, and linezolid) for multi-drug resistant pulmonary tuberculosis [7]; a regimen that shortens pulmonary tuberculosis treatment to 4 months [8], and innovative phase 2 trials that will speed the passage of more new drugs into phase 3 trials and clinical practice [9]. There is also growing interest in novel adjunctive therapies that target specific components of tuberculosis inflammation linked to substantial long-term morbidity in survivors [10].

It is time for TBM research to catch up. The panoply of new and emerging antimicrobial and adjunctive anti-inflammatory agents offers an unparalleled opportunity to reduce TBM morbidity and mortality. If we continue to conduct small trials, testing one intervention at a time, with an inflexible and poorly coordinated approach, our patients will be no better off in 10 years. A new TBM clinical trials platform is needed, which will enable the efficient testing of new drugs and regimens as they emerge from phase 2 trials, running in parallel with the trials conducted in pulmonary tuberculosis.

We, therefore, propose a global, phase 3, adaptive, 2-stage randomized controlled platform trial that will investigate multiple interventions to reduce death and disability from TBM, at scale and at speed. The trial platform will be built across Asia and Africa because this is where most of the disease and the expertise to tackle it resides. We will coordinate with the ‘Tuberculous Meningitis International Research Consortium’, a 15-year-old global community of TBM researchers, to which the authors belong, to ensure that we include the world's leading TBM investigators and trial sites. The design and delivery of the trial, and the uptake of the results, will depend upon the strength of this global consortium, the trust between members and their substantial external influence.

The trial will be as pragmatic as possible, while not compromising on rigour and quality. The trial must have the capacity to investigate new anti-tuberculosis or anti-inflammatory drugs, therefore must be able to support submissions to regulatory agencies. The pragmatism, therefore, will come from broad inclusion criteria, reducing unnecessary barriers to participation, thus ensuring real-world relevance of the results, and by the use of easily ascertained outcomes that matter to patients. We envisage a modular protocol, with a ‘core’ component for all sites that articulates the essential elements of the trial, with the optional addition of modules (e.g. pathophysiology and pharmacology) for the research-intensive centres.

In the first stage of the platform's activities (years 1−4), there will be 2 main objectives. The first will be to identify one or more 26-week regimens either non-inferior or superior to the current WHO-recommended 52-week standard-of-care regimen, which would then become the new ‘standard-of-care’ in stage 2 of the platform. A stringent non-inferiority margin would be required (relative risk of at most 10% increased mortality or disability). At least one of these regimens should be rifampicin-free. A regimen for rifampicin-resistant TBM is urgently needed, and rifampicin has extensive drug-drug interactions, complicating co-administration and hindering the development of novel, highly active regimens containing new drugs. Bedaquiline-based regimens have fewer interactions than rifampicin-based regimens and are highly effective in treating multi-drug resistant tuberculosis, making them an attractive backbone for new TBM regimens. The second objective will be to determine whether enhanced early immunomodulation, with higher corticosteroid doses, aspirin, or tumour necrosis factor (TNF)-α antagonists (e.g. infliximab), is superior to the current standard-of-care dexamethasone. In the second stage (years 5–10), the platform would substitute new anti-tuberculosis and anti-inflammatory drugs emerging from phase 2 trials into the new ‘standard-of-care’ regimens identified in stage 1, thereby seeking to further reduce death and disability from TBM. Decisions to replace the current standard-of-care would be based on showing superiority.

The interventions tested by the trial in stage 1 (including the drug choices and doses) need discussion and agreement with the TBM consortium trial investigators and industry collaborators where appropriate. Intervention choices may also be informed by animal models [11]. As in previous trials, the interventions will subdivide into those that enhance bacterial killing, and those that control brain inflammation. Both can be hypothesized to reduce death and neurological disability from TBM, with the advantage that their limited or absent interaction enables a factorial trial design (Fig. 1).

The trial will enrol anyone with suspected TBM, regardless of HIV status. The trial may start in adults, but TBM is common in children, often with devastating outcomes, and extension into all age groups should be a priority. We and others in the TBM consortium are investigators in the active, multi-centre (Asia and Africa), SURE trial in childhood TBM (ISRCTN40829906), providing an infrastructure for including children in the proposed trial. Intervention-specific exclusion criteria are inevitable but will be kept to a minimum. The primary outcome will be death or disability, assessed monthly in person or by telephone using the modified Rankin score, up to 52 weeks after randomization [12]. Secondary outcomes will be limited to the common inflammatory intra-cerebral complications of treatment (so-called paradoxical reactions or immune reconstitution inflammatory syndrome in those initiating anti-retroviral treatment), drug-related serious adverse events, treatment failure, and relapse.

To enable the efficient selection of the regimens most likely to improve upon the current standard-of-care, we will use a multiarm adaptive design. An independent data monitoring committee will make regular pre-specified comparisons of death and disability and drug-related adverse events in the intervention arms relative to the standard-of-care. Poorly performing interventions can be dropped for futility. The arms predicted most likely to improve upon the standard-of-care will continue to enrol. The majority (>80%) of deaths in TBM occur within 2 months of treatment initiation, allowing for rapid ascertainment of most end points. This allows for regular interim analyses using patient data right censored at the most recent follow-up. Improvements in the current standard-of-care would most likely be moderate (approximately 20% reduction in death or disability). In stage 1, showing non-inferiority or superiority of a marginally better regimen (10% reduction in mortality or more) would require around 500 patients per intervention under a non-inferiority margin of 4 percentage points. With the engagement of the TBM consortium trials community, we anticipate recruiting at least 500 participants each year.

Intra-cerebral inflammatory reactions occur in approximately 20% of patients, usually 4 to 12 weeks after starting TBM treatment. They include the expansion of space-occupying lesions (tuberculomas) and vasculitis with multiple infarcts. Approximately 30% of these reactions result in death or disability; however, their management has never been subject to randomized controlled trials. Generally, corticosteroids are given (e.g. dexamethasone), but the optimal dose and duration are unknown [13]. Targeted anti-inflammatory drugs are hypothesized to be safer and more effective than corticosteroids, but support for their use is limited to case series. These drugs include antagonists of the cytokines TNF-α (e.g. infliximab and thalidomide) and interleukin (IL)-1 β (e.g. anakinra) [14,15].

Given the ongoing uncertainty surrounding the management of the inflammatory reactions and the lack of relevant trial data, trial participants experiencing these events (defined clinically and radiologically) will be eligible for third randomization (Fig. 1). We will likely compare 2 interventions initially–high-dose corticosteroids and infliximab–with the primary outcome being death or disability 12 months from the baseline randomization. As approximately 20% of trial participants will be eligible, and the anticipated effect sizes on the primary outcome are unknown, this part of the trial would be exploratory. We will take advantage of the platform design to continue randomization in stage 2, until such time as the data monitoring committee recommends stopping based on effi-cacy, safety, or futility.

In summary, we propose a global, multi-arm, two-stage, adaptive randomized controlled platform trial that will accelerate the testing of new anti-tuberculosis and anti-inflammatory drugs for the optimal treatment of TBM. We envisage the trial being closely linked to the innovative phase 2 and 3 trials of new drugs now being undertaken and planned for pulmonary tuberculosis treatment. Early data sharing and knowledge integration between these trials will be essential if they are to lead to a substantial fall in global tuberculosis morbidity and mortality.

## Figures and Tables

**Fig. 1:**
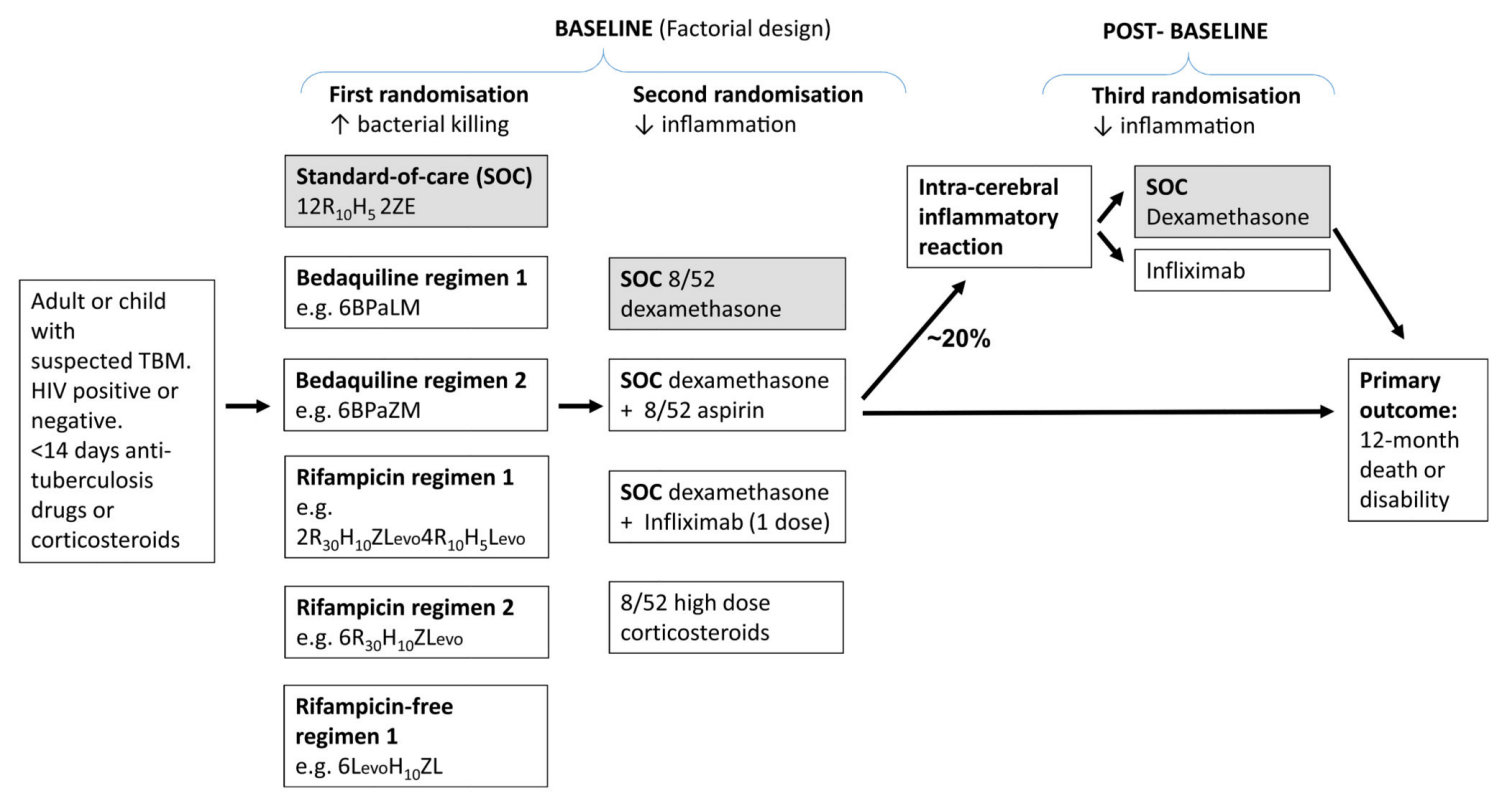
Participant flow for stage one of a global, adaptive, randomized controlled platform trial the best anti-tuberculosis and anti-inflammatory treatment of TBM. Details of the interventions/regimens will be decided by the trial investigators. Doses of anti-tuberculosis drugs will be adjusted according to age. For the proposed regimens: B, bedaquiline; H, isoniazid; L, linezolid; Levo, levofloxacin; M, moxifloxacin; Pa, pretomanid; R, rifampicin; Z, pyrazinamide. The larger numbers indicate the duration in months; the subscript numbers indicate the dose (mg/kg/d).
